# Enhancing Breast Cancer Screening Uptake: A Community-based Intervention in Southeast Wolverhampton, UK

**DOI:** 10.7759/cureus.87788

**Published:** 2025-07-12

**Authors:** Obinna Onyedum, Parpinder Singh, Tsz Ki Tan, Ajith Kumar Kannan, Salma Reehana, Rajnish Mohindroo, Rahul Mittal

**Affiliations:** 1 Health Informatics, Rutgers University, Piscataway, USA; 2 Public Health, City of Wolverhampton Council, Wolverhampton, GBR; 3 Internal Medicine, Zhongshan School of Medicine, Guangzhou, CHN; 4 General Practice, Health and Beyond Partnership, Wolverhampton, GBR

**Keywords:** breast cancer screening, health inequalities, invitation uptake, nhs, oncology, preventive health promotion, public health

## Abstract

Objectives

Breast cancer screening rates in Wolverhampton, UK, have fallen below national targets. This study examines potential contributing factors to low screening uptake and evaluates whether collaboration between primary care networks (PCNs) and public health initiatives is associated with improved screening participation and health promotion efforts. The research focuses on Wolverhampton Southeast PCN, assessing the effectiveness of a joint approach in addressing health inequalities and enhancing targeted outreach.

Setting and methods

A targeted intervention was implemented to re-engage women who had previously missed breast cancer screening appointments. The intervention employed a multi-faceted strategy, incorporating high-quality telephone consultations and a self-booking system. The study involved two primary care practices in Wolverhampton - Bilston Urban Village and Bilston Medical Centre. Eligible participants were identified based on prior non-attendance, with 713 women included in the study.

Results

The intervention led to a 22% screening uptake (126 of the 581 invited participants) among 713 women who had previously not attended their breast screening appointments, demonstrating the potential of targeted strategies to improve participation. However, screening rates remain below the National Health Service (NHS) minimum target of 70%, indicating the need for further tailored interventions.

Conclusion

The findings underscore persistent disparities in breast cancer screening uptake within Wolverhampton's primary care network. While targeted interventions can enhance engagement, additional efforts are required to bridge the gap and achieve national screening targets. Strengthening collaboration between primary care and public health may be key to addressing health inequalities and improving preventive health measures.

## Introduction

Breast cancer continues to pose a significant global health burden, particularly among women. In 2022, an estimated 2.3 million women were diagnosed with breast cancer worldwide, leading to approximately 670,000 deaths [1]. In the UK, breast cancer is the most frequently diagnosed cancer, representing 15% of all new cancer cases between 2017 and 2019. Each year, around 56,800 new cases are recorded - equivalent to over 150 diagnoses daily - resulting in approximately 11,500 deaths annually [2].

Timely diagnosis remains a cornerstone in improving breast cancer outcomes. Screening, especially through mammography, is central to early detection efforts and is associated with substantial reductions in mortality. Evidence suggests that regular participation in mammography screening can reduce the risk of breast cancer death by up to 35% [3,4]. In the UK, the National Health Service Breast Screening Programme (NHSBSP), established in 1988, invites women aged 50 to 70 to undergo routine mammographic screening every three years [5,6]. While the program has contributed significantly to earlier detection and reduced mortality, participation rates vary considerably across regions.

Wolverhampton, in particular, has consistently demonstrated lower screening uptake than both national and regional averages. According to 2024 figures from the Office for Health Improvement and Disparities (OHID), the screening rate in Wolverhampton was 57%, compared to 66.2% nationally and 63.6% across the broader Black Country region [7]. Furthermore, Wolverhampton has the highest breast cancer mortality rate among the four Black Country local authorities [8,9]. These disparities underscore the urgent need for targeted interventions to improve screening participation and reduce preventable deaths.

Barriers to screening are multifactorial and include cultural beliefs, stigma, logistical challenges, and socioeconomic disadvantage [10,11]. While various strategies, such as modified invitation methods and community-based awareness campaigns, have been implemented to address these issues, many have achieved only limited success [12-14]. In particular, there remains a gap in approaches that prioritize direct, personalized communication between patients and healthcare professionals as a mechanism for improving screening engagement [15,16].

In response to these challenges, a community-based intervention was co-developed through collaboration between the Wolverhampton local authority Public Health team, the Southeast Primary Care Network, the Integrated Care Board Black Country, and the regional breast screening service. The initiative focused on re-engaging women, who had previously missed scheduled screening appointments, using tailored outreach and enhanced patient-provider interaction to address both structural and interpersonal barriers to participation.

This study aimed to assess whether this collaborative intervention could improve breast cancer screening uptake among women who had previously not attended their appointments. Additionally, we examined demographic factors - including age, ethnicity, and socioeconomic status - that may influence participation. A successful outcome was defined as a measurable increase in screening uptake relative to pre-intervention baseline.

## Materials and methods

Study design and overview

This intervention utilized a multi-faceted, practice-based approach to re-engage women who had missed scheduled breast cancer screening appointments. Central to the intervention was a personalized, high-quality telephone conversation designed to educate, address concerns, and facilitate rebooking. The project was implemented in partnership with the Southeast Collaborative Primary Care Network (SE PCN), which identified two local practices with recent breast screening activity and significant numbers of non-attendees. These practices - Bilston Urban Village and Bilston Medical Centre - served as pilot sites for the intervention.

Ethical considerations

This project was undertaken as a service improvement and evaluation initiative arising from routine primary care quality assurance activities. In line with national guidance, ethical approval was not required [17]. No patient-identifiable data were collected or used; all analyses were conducted on anonymized, aggregate screening data. The project posed minimal ethical risk and adhered to established standards for audit and quality improvement.

Participating practices and target population

*Bilston Urban Village Practice*: Situated in the South Ward of Bilston, this practice serves approximately 13,000 patients and is located in a highly deprived area (deprivation decile 2, with 1 being most deprived). The patient population is ethnically diverse: 75.2% White, 13.9% Asian, 5.2% Black, 4.5% mixed, and 1.2% from other ethnic backgrounds. The practice operates from 8 a.m. to 8 p.m. on weekdays and offers weekend access from 8 a.m. to 12 p.m. Historical uptake for breast cancer screening was 47%.

*Bilston Medical Centre*: Located in the Bilston North Ward, this smaller practice has approximately 3,600 patients and serves a highly deprived demographic, particularly affected by income deprivation among children and older adults. The practice population includes a larger proportion of older patients. Prior screening uptake for this site was 59%.

Eligibility criteria

Participants included women aged 50-70 years who had failed to attend their most recent breast cancer screening appointments. Eligible individuals were identified using the Systematized Nomenclature of Medicine - Clinical Terms (SNOMED CT) codes that indicated missed appointments and were cross-referenced with breast screening service data to confirm eligibility. Women who had officially opted out of the NHS Breast Screening Programme (indicated by an ‘opt-out’ SNOMED CT code) were excluded from the study. A total of 713 women were identified as non-responders: 542 from Bilston Urban Village and 171 from Bilston Medical Centre.

Intervention focus

**Figure 1:**
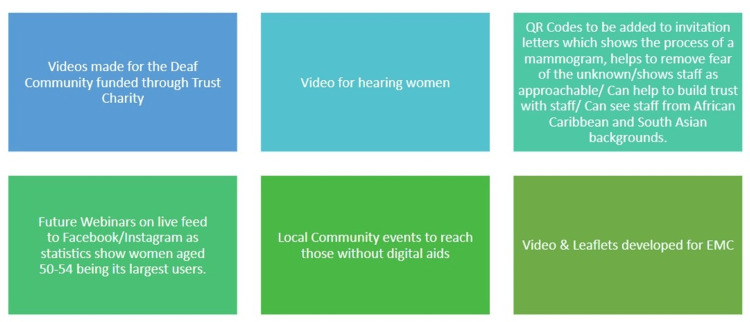
Key initiatives emphasized during the refresher training session for staff to improve patient engagement and screening uptake.

1. *Quality Conversations*: Primary care staff participated in a refresher training session to reinforce previously acquired skills in cancer screening communication. The session was co-developed with the local Integrated Care Board Cancer facilitator and covered key topics including motivational interviewing techniques, culturally sensitive language, common myths and misconceptions about mammography, and strategies for handling emotional resistance. Printed job aids and conversation guides published by trusted sources such as the NHS and Cancer Research UK were provided to support consistency. Staff were trained to identify and address individual barriers to screening, raise awareness of cancer symptoms, and offer culturally competent explanations of the mammography process. Practice-branded, personalized invitation calls served as a critical component of patient re-engagement, intended to build trust and encourage action.

2. *Self-Booking System*: Although not part of the initial intervention plan, a self-booking option emerged as a key innovation. Leveraging the Accurx texting platform, practices enabled patients to schedule their screening appointments directly [18]. The system was configured to generate a unique link for each patient, directing them to a booking interface aligned with appointment slots released by the Breast Screening Service. Patients could access the system by entering their demographic details, such as date of birth and postcode. This approach enabled patients to select a convenient appointment time, enhancing flexibility and reducing barriers associated with traditional booking methods. It also streamlined administrative processes, as confirmed bookings could be easily tracked and communicated to the clinic team, minimizing the need for manual coordination.

3. *Community Engagement and Patient Interaction Enhancement*: To overcome broader accessibility barriers and improve patient engagement, the participating practices implemented a series of targeted, community-oriented initiatives (Figure 1). Informational videos were developed specifically for the deaf community to ensure inclusivity and better understanding of the screening process. To reach individuals without digital access, local community events were organized, providing in-person opportunities for engagement and education. Additionally, future webinars were planned for dissemination via social media platforms that are popular among women aged 50-54 years, aiming to extend outreach through familiar and accessible digital spaces. To further personalize the invitation process, letters included quick response (QR) codes linking to short videos that demonstrated the mammography procedure and featured diverse staff members. These visual aids were designed to demystify the screening experience, reduce anxiety, and foster trust in the screening service.

Mobile screening accessibility

The intervention utilized a mobile mammography unit stationed at a large supermarket car park in Bilston, Wolverhampton. This location, operated by the regional Breast Screening Service (Dudley, Wolverhampton, and Southwest Staffordshire), was chosen for its accessibility and visibility within the local community. The unit was temporarily deployed during the study period to improve access to breast screening services for women in underserved areas.

Data collection and analysis

Data were collected longitudinally from the breast screening service and participating practices throughout the intervention. Individual-level information on age, ethnicity, and index of multiple deprivation was captured. Additionally, data were compiled regarding the number of individuals contacted, appointments scheduled, and screening attendance rates. While outcome data were also obtained from these sources, the quality and completeness of the data were negatively impacted by staffing shortages.

All data were securely stored in Microsoft Excel (Microsoft, Redmond, WA) on encrypted NHS devices. The dataset was reviewed for consistency, and any discrepancies were resolved through consultation with practice managers. The results are expressed as numbers and percentages. Given the small study population, we employed descriptive statistics to summarize key variables and explore possible patterns or associations. No inferential statistics were applied due to the limited sample size and non-randomized design.

## Results

Data availability and inclusion

This analysis aimed to include all patients who were contacted (i.e., offered a screening), those who subsequently booked an appointment, and those who ultimately attended a breast screening session. Demographic data were fully available from one participating practice - Bilston Urban Village - which enabled stratified analysis across these three patient engagement domains.

However, due to patient record coding issues at Bilston Medical Centre, demographic data for patients who attended screening were not available and were therefore excluded from exploratory demographic analysis. Additionally, postcode data for patients from Bilston Medical Centre who booked a screening appointment were missing and subsequently omitted from the study. A summary of data inclusion and exclusion is provided in Table 1.

**Table 1:** Summary of data used for the exploratory data analysis

		Bilston Urban Village (BUV)	Bilston Medical Centre (BMC)
Screening Offered (Contacted)	Age	Yes	Yes
	Ethnicity	Yes	Yes
	Postcode	Yes	Yes
Appointments booked as a result of contact	Age	Yes	Yes
	Ethnicity	Yes	Yes
	Postcode	Yes	No
Screening appointment attended and completed because of the booking	Age	Yes	No
	Ethnicity	Yes	No
	Postcode	Yes	No

Screening invitation and attendance rates

Out of 713 previous non-responders identified, 581 individuals (82%) were successfully contacted and invited to participate in the NHS Breast Screening Programme. Of those invited, 193 women (33%) booked a screening appointment, and among these, 126 (65%) attended their scheduled session. This equates to an overall screening attendance rate of 22% across all invited patients. The Breast Screening Service reported a total of 126 appointments completed, which was used for final analysis to minimize the risk of discrepancies in practice-reported data. Bilston Urban Village accounted for 62 confirmed attendances, while final attendance data for Bilston Medical Centre was not available (Table 2).

**Table 2:** Summary of activity from the project at Wolverhampton. Breast Screening Service reported that 713 patients did not attend their screening (DNA); 581 patients were contacted and among them, 193 booked appointments; this number has been used for the analysis rather than the practice reported to minimize the risk of data error. Attendance data was unavailable from BMC. BUV reported that 62 of their patients attended the screening. Breast Screening Service reported 126 patients attended for screening overall. Data reported by the screening service has been used for attendance to minimize the risk of data error.

Practice	DNA Cohort	Contacted	Booked	Attended
Bilston Urban Village (BUV)	542	438	112	62
Bilston Medical Centre (BMC)	171	143	43	Not provided

Exploratory demographic analysis (Bilston Urban Village Only)

**Figure 2:**
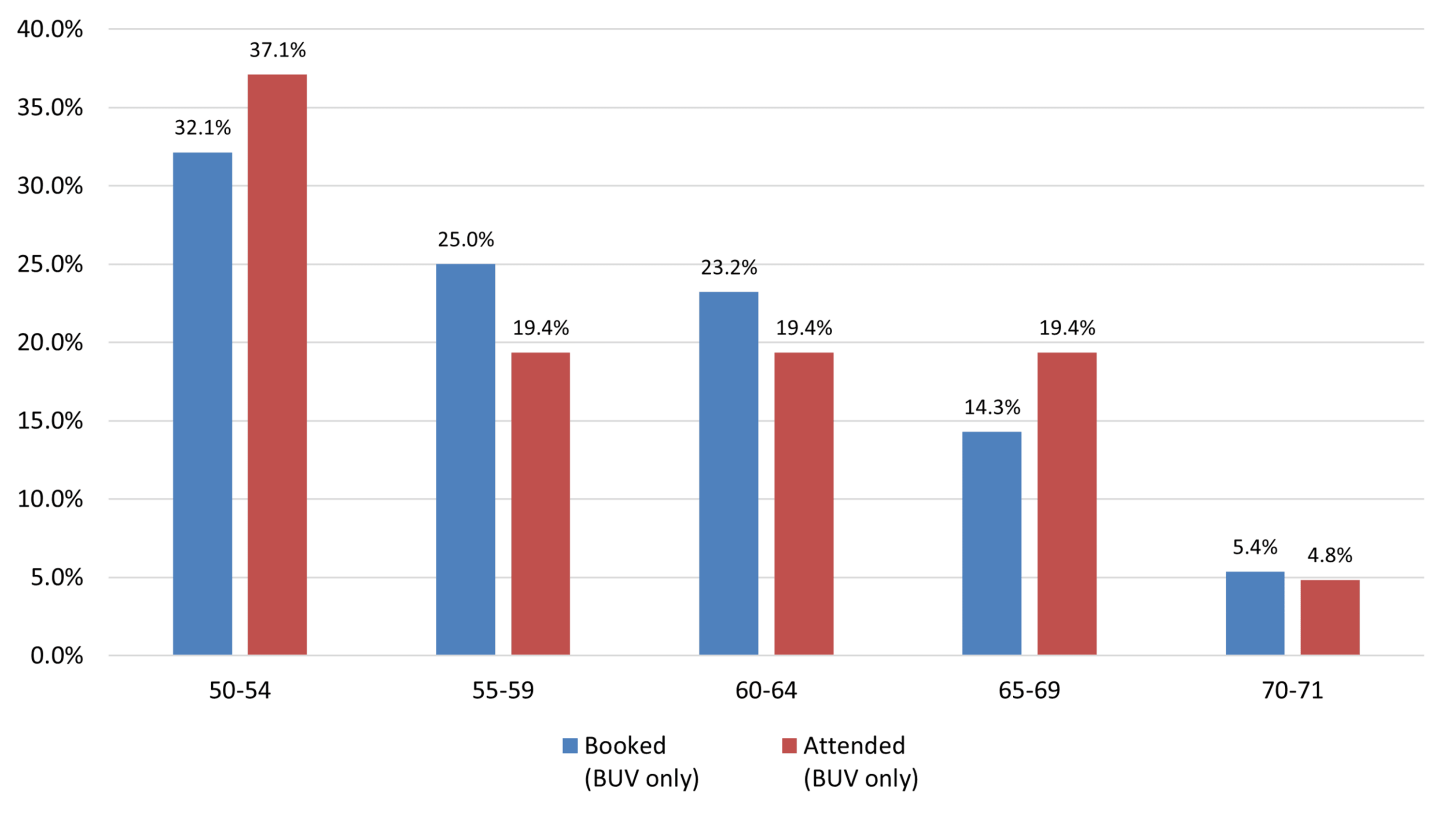
Age group comparison of Patients booked an appointment compared to those who attended at Bilson Urban Village (BUV)

*Age*: Analysis by age group revealed that patients aged 50-54 years demonstrated the highest screening uptake following invitation, while those aged 65-69 had the lowest uptake. Among patients who booked a screening appointment, the 55-59 age group exhibited the lowest attendance rate (Figure 2).

The national breast screening programme in England officially invites individuals up to the age of 70. However, there can occasionally be a small number of individuals screened after the age of 70. This typically happens in cases where someone receives their invitation just before turning 70 but attends their appointment after their birthday. As a result, while there may be some screening activity recorded for those over 70, the numbers are naturally very low and should be interpreted in that context. This is expected and aligns with how the programme operates.

**Figure 3:**
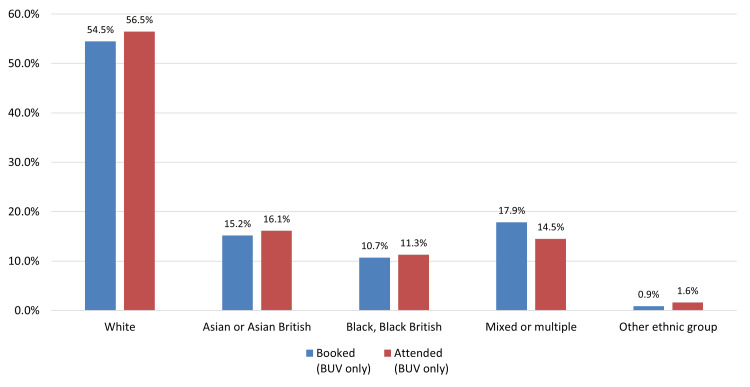
Ethnicity Comparison of Patients offered screening (contacted) compared to those who booked an appointment at Bilston Urban Village (BUV)

*Ethnicity*: Caucasian (White) patients showed the highest levels of screening uptake after invitation, while those identified as “Other ethnic groups” had the lowest. Of those who booked, the “Mixed or multiple ethnic groups” category had the lowest attendance rate, whereas the Caucasian group had the highest (Figure 3).

**Figure 4:**
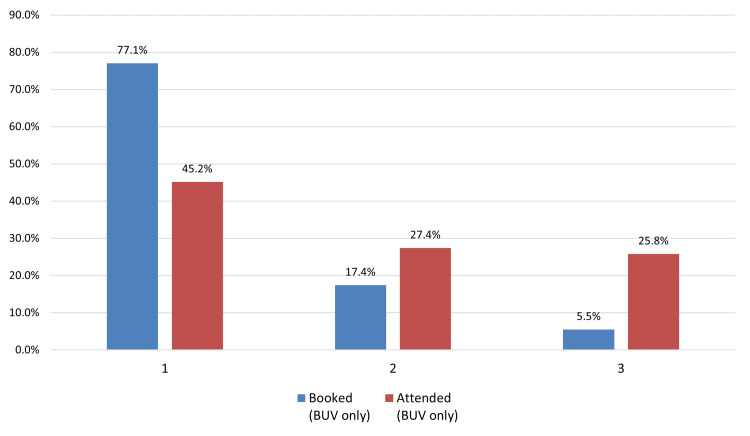
Deprivation of area comparison for Patients who booked an appointment compared to those who attended at Bilston Urban Village (BUV)

*Deprivation (Index of Multiple Deprivation (IMD))*:* *Deprivation levels were categorized using IMD quintiles based on the location of general physician (GP) surgeries. The most deprived quintile (IMD 1) demonstrated the highest uptake following screening invitation, while IMD 3 had the lowest uptake. Interestingly, among those who booked appointments, patients in IMD 3 had the highest attendance rate, while IMD 1 had the lowest (Figure 4).

**Figure 5:**
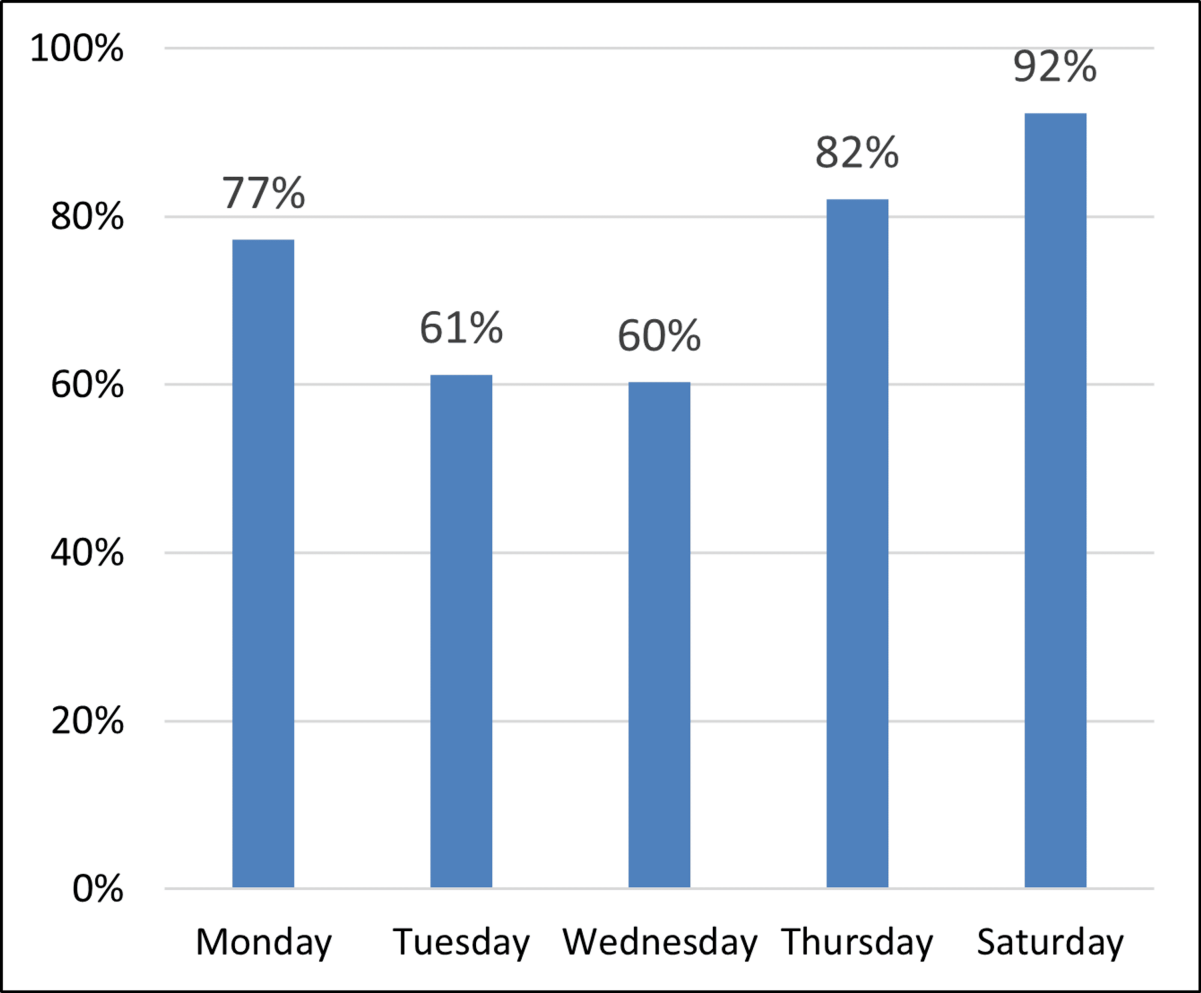
Percentage of Appointments slots available against slots filled at Bilston Urban Village (BUV)

*Day of the week*: Screening activity data indicated a modest preference for later weekdays, particularly Thursdays and Saturdays. A single Saturday clinic with 13 available slots demonstrated the highest proportional attendance, with 12 of the 13 slots filled (Figure 5).

Qualitative insights

Feedback from staff involved in the intervention identified the self-booking system as a key facilitator of patient engagement. The capability for patients to autonomously schedule appointments using a user-friendly, text-based platform (Chain SMS, Accurx, London, UK) was reported to significantly improve both accessibility and compliance [18].

While no direct staff quotations were formally recorded, observations from implementation meetings and staff discussions consistently pointed to the system’s ease of use and its role in reducing administrative burden. These reflections informed the interpretation that the self-booking system contributed positively to patient uptake.

## Discussion

This study investigated disparities in breast cancer screening uptake within the Wolverhampton Primary Care Network and demonstrated the potential of personalized, community-based interventions to improve screening adherence among historically disengaged populations. While the observed attendance rate of 22% remains below the NHS minimum target of 70%, the findings affirm the value of proactive, empathetic patient outreach as an effective re-engagement strategy. Nevertheless, because follow-up was limited to the immediate post-intervention period, the durability of these gains across future screening cycles is unknown; sustained benefit will require ongoing monitoring and reinforcement.

Consistent with existing literature, age-based disparities were observed, with lower screening participation among women aged 65-69 years compared to younger age groups. Taplin and Montano (1993) previously identified reduced awareness among older women regarding the benefits of mammography in detecting asymptomatic cancers, underscoring the need for age-sensitive messaging to support informed participation in later life [19]. Ethnic inequalities were also evident. Women from non-White backgrounds, particularly those categorized under "Other ethnic groups," exhibited the lowest participation. Prior studies have shown similar patterns, with Jack et al. (2014) reporting significantly lower uptake among Black, Asian, and minority ethnic (BAME) women in the UK [20]. These disparities are often rooted in cultural stigma, language barriers, and structural distrust, which collectively hinder engagement. Socioeconomic disadvantage further compounds screening non-adherence. As shown by Douglas et al. (2016) and von Wagner et al. (2011), individuals from deprived backgrounds encounter multiple logistical barriers, including limited transportation, inflexible work schedules, and lack of childcare [21,22]. Our findings mirrored these challenges, particularly in Wolverhampton’s most deprived areas, despite initial positive responses to outreach invitations.

Globally, community-centered approaches have emerged as promising strategies to bridge the gap in cancer screening. Effah et al. (2025) documented the success of a sustainable, trainer-led outreach program in Ghana that integrated cervical and breast cancer screening into localized healthcare infrastructure [23]. Similarly, Christie-de Jong et al. (2022) demonstrated that a culturally adapted, faith-based intervention targeting Muslim women in Scotland enhanced screening engagement through peer-led discussions and religious framing [24]. These insights align with Moyce et al. (2024), who employed visual participatory tools-such as fishbone diagrams, mind maps, and concept mapping-in collaboration with Hispanic women in rural Montana [25]. Their study identified key barriers, including communication gaps, inflexible scheduling, a lack of bilingual providers, and transportation difficulties. Their participatory process exemplifies how research can be democratized to co-create localized, scalable solutions. In a complementary vein, Kasper et al. (2024) provided a comprehensive analysis of individual and neighborhood-level socioeconomic determinants of breast cancer screening adherence [26]. Using high-dimensional modeling, they found that housing insecurity, food insecurity, and neighborhood overcrowding were independently associated with screening non-adherence. Their work confirms the broader applicability of the fundamental cause theory, which suggests that structural inequalities, rather than individual behavior alone, shape health outcomes. It further underscores the importance of embedding social determinants into intervention planning to avoid perpetuating disparities. Strategies such as flexible appointment systems, mobile units, peer navigation, multilingual resources, and integration with local faith or community leaders may mitigate barriers and improve trust [26]. Moreover, incorporating socioeconomic indicators, such as renting status or food insecurity, into risk stratification tools may help target those at highest risk of non-participation.

Ultimately, the Wolverhampton case study offers an example of how contextually grounded, relational approaches can enhance cancer prevention efforts. Future research should incorporate longitudinal follow-up to determine whether initial improvements are maintained and to identify factors that support sustained engagement. Additional studies should also assess scalability across diverse demographic and geographic settings.

Limitations

Several limitations were encountered during the course of this study, which constrained the depth and generalizability of the findings. First, the study focused on a relatively small sample size of 713 non-responders. A larger sample, incorporating a more diverse cross-section of women across various sociodemographic backgrounds, would strengthen the validity and generalizability of the results. Second, incomplete data from Bilston Medical Centre - particularly the absence of attendance figures - limited the ability to draw comparisons across participating practices and to assess overall intervention effectiveness within the primary care network. Third, although qualitative feedback about the self-booking system was positive, broader qualitative data were limited. More comprehensive feedback from participants regarding barriers to screening and their motivations for attendance or non-attendance would have enriched the analysis and offered deeper insight into behavioral drivers.

Additionally, the study employed only short-term follow-up, focusing on immediate uptake and attendance post-intervention. Long-term adherence to routine breast screening intervals remains unexplored, yet is critical to understanding sustained behavioral change. There is also a potential for selection bias, as individuals who responded to the intervention may differ in motivation, health literacy, or other unmeasured factors from those who remained disengaged. This limits the ability to generalize the findings to all non-attenders. Furthermore, the study was not designed or powered to support inferential statistical analysis; as such, conclusions about associations between demographic characteristics and screening uptake should be interpreted with caution.

Despite these constraints, the limitations also identified key areas where future interventions and research efforts can be directed.

## Conclusions

Improving breast cancer screening rates requires tailored, multi-faceted strategies that address diverse population needs. This intervention demonstrated potential but highlights ongoing gaps to be addressed. Building on successes like flexible scheduling and self-booking can drive further progress toward national screening targets.

## References

[1] Who. (2024 (2025). World Health Organization. Breast cancer. https://www.who.int/news-room/fact-sheets/detail/breast-cancer.

[2] Cancer Research, U.K. (2024 (2025). Cancer Research UK. Breast cancer statistics. https://www.cancerresearchuk.org/health-professional/cancer-statistics/statistics-by-cancer-type/breast-cancer#heading-Zero.

[3] International Agency for Research on Cancer (2025). Breast cancer screening. I. ARC Handbooks of Cancer Prevention, Volume 15.

[4] Advisory Committee on Breast Cancer Screening (2006). Screening for breast cancer in England: past and future. J Med Screen.

[5] Gov.Uk. (2024 (2025). Gov.uk. Breast screening: programme overview. https://www.gov.uk/guidance/breast-screening-programme-overview.

[6] Hogben RK (2008). Screening for breast cancer in England: a review. Curr Opin Obstet Gynecol.

[7] Office For Health (2025). Office for Health Improvement and Disparities. Public Health Profiles - OHID. https://fingertips.phe.org.uk/search/breast%20screening#page/1/gid/1/pat/204/par/U26715/ati/7/iid/91339/.

[8] Breast Cancer Now. (2023 (2025). Breast Cancer Now. Barriers to breast cancer screening. https://breastcancernow.org/about-us/campaign-news/barriers-breast-cancer-screening/.

[9] Vrinten C, Gallagher A, Waller J, Marlow LA (2019). Cancer stigma and cancer screening attendance: a population based survey in England. BMC Cancer.

[10] Woof VG, Ruane H, Ulph F (2020). Engagement barriers and service inequities in the NHS Breast Screening Programme: views from British-Pakistani women. J Med Screen.

[11] Mottram R, Knerr WL, Gallacher D (2021). Factors associated with attendance at screening for breast cancer: a systematic review and meta-analysis. BMJ Open.

[12] Kearins O, Walton J, O'Sullivan E, Lawrence G (2009). Invitation management initiative to improve uptake of breast cancer screening in an urban UK Primary Care Trust. J Med Screen.

[13] Hudson S, Brazil D, Teh W, Duffy SW, Myles JP (2016). Effectiveness of timed and non-timed second appointments in improving uptake in breast cancer screening. J Med Screen.

[14] Allgood PC, Maroni R, Hudson S (2017). Effect of second timed appointments for non-attenders of breast cancer screening in England: a randomised controlled trial. Lancet Oncol.

[15] Boothby C, Patterson K, Premji S (2023). Creating health equity in cancer screening: developing outreach strategies for under-screened populations through community engagement. Healthc Q.

[16] Khattak HM, Woof VG, French DP (2023). The role of knowledge, primary care and community engagement to improve breast-screening access for Pakistani women in the United Kingdom: a secondary analysis of a qualitative study. J Health Serv Res Policy.

[17] (2025). What approvals and decisions do I need?. https://www.hra.nhs.uk/approvals-amendments/what-approvals-do-i-need/.

[18] Mittal R, Kannan AK, Mohindroo R, Movva C, Zhang L, Reehana S, Srinivasan S (2025). Implementing Accurx for total triage enhancing care navigation and patient experience. Cureus.

[19] Taplin SH, Montano DE (1993). Attitudes, age, and participation in mammographic screening: a prospective analysis. J Am Board Fam Pract.

[20] Jack RH, Møller H, Robson T, Davies EA (2014). Breast cancer screening uptake among women from different ethnic groups in London: a population-based cohort study. BMJ Open.

[21] Douglas E, Waller J, Duffy SW, Wardle J (2016). Socioeconomic inequalities in breast and cervical screening coverage in England: are we closing the gap?. J Med Screen.

[22] von Wagner C, Good A, Whitaker KL, Wardle J (2011). Psychosocial determinants of socioeconomic inequalities in cancer screening participation: a conceptual framework. Epidemiol Rev.

[23] Effah K, Tekpor E, Wormenor CM (2025). A community-focused cervical and breast cancer screening program using a sustainable funding model in a training center in Ghana. BMC Health Serv Res.

[24] Christie-de Jong F, Kotzur M, Amiri R, Ling J, Mooney JD, Robb KA (2022). Qualitative evaluation of a codesigned faith-based intervention for Muslim women in Scotland to encourage uptake of breast, colorectal and cervical cancer screening. BMJ Open.

[25] Moyce S, Comey D, Claudio D, Velazquez M, Reyes GC, Aghbashian E (2024). A community-engaged approach to reducing barriers to breast and cervical cancer screening: the use of mapping tools. J Particip Res Methods.

[26] Kasper G, Momen M, Sorice KA (2024). Effect of neighborhood and individual-level socioeconomic factors on breast cancer screening adherence in a multi-ethnic study. BMC Public Health.

